# Biology, Ecology, Distribution and Control of the Invasive Weed, *Lactuca serriola* L. (Wild Lettuce): A Global Review

**DOI:** 10.3390/plants10102157

**Published:** 2021-10-11

**Authors:** Aakansha Chadha, Singarayer Florentine

**Affiliations:** Future Regions Research Centre, School of Science, Psychology and Sport, Federation University Australia, Mount Helen, VIC 3350, Australia; s.florentine@federation.edu.au

**Keywords:** wild lettuce, prickly lettuce, invasive species, invasive alien species

## Abstract

*Lactuca serriola* L. (wild lettuce) is a highly invasive C_3_ weed in many countries, including Australia, Canada, and the USA. This weed is a severe threat to agricultural systems, especially in crops grown with reduced or no-tillage approaches, which commonly include wheat, cereals and pulses. Owing to the vertical orientation of its leaves in the north-south plane and its root architecture, *L. serriola* can maintain high water use efficiency under drought conditions, giving it the ability to expand its range under a drying climate. Each plant can produce up to 100,000 seeds which have no primary dormancy and form a short-term seedbank lasting up to three years. Most seedlings emerge in autumn and overwinter as a rosette, with a small flush of emergence in spring depicting staggered germination. Research into control methods for this weed has been performed, and these methods include chemical herbicides applied alone and in combination, the establishment of plant competition, tillage, mowing and bioherbicide. Herbicides can provide effective control when applied in the seedling or rosette stage; however, spring germination is difficult to control, as it skips the rosette stage. Some biotypes are now resistant to ALS inhibitor and synthetic auxins, causing concern regarding using herbicides. A dedicated integrated management plan for 3–4 years is recommended for the control of this troublesome species. This review will explore the biology, ecology, distribution, current control techniques and previous research on this weed, allowing us to make recommendations for its future research and management.

## 1. Introduction

The genus *Lactuca* originated in the Mediterranean Basin, with *Lactuca serriola* L. being the most common and widely distributed species of this genus [1,2]. The genus name, *Lactuca*, incorporates the Latin word “lac” for milk, referring to the milky sap produced by the plant. *L. serriola* is commonly known as prickly lettuce, wild lettuce or compass plant, and belongs to the family Asteraceae. It featured in an assessment of the top 20 national residual weeds in Australian agriculture, affecting the yield and revenue return of canola, pulses and winter cereals crops [3]. It is spread over 77,500 hectares in the southern part of the continent, with a recent review indicating that there is a yield loss of 2979 tons and revenue loss of approximately AUD 730,000 caused annually by this weed [3]. In addition, it has also been observed that this species has become resistant to some of the ALS-inhibiting herbicides in South Australia, which makes its control increasingly difficult [3,4].

*Lactuca serriola*, a C_3_ species, is also a significant agricultural and environmental weed in many countries other than Australia. It is mostly distributed on roadsides, grassy ditches, dust-heaps and ruderal areas, and is also found as weed communities in agricultural crops and native pastures [5]. *L. serriola* can reach a height of 2 m and, owing to its deep tap root system, is a tough competitor for growing crops or pastures [6]. By competing for water, nutrients, space, and light, this species affects crop yields, and if left unchecked, it aggressively consumes soil moisture and nutrients, especially in the summer. According to studies, *L. serriola* causes yield losses of up to 10% at low densities of 0.2–1.2 plants m^−2^ and up to 80% at concentrations greater than 50 plants m^−2^ [7].

In addition to yield loss, this weed species is also known to reduce grain quality and harvesting efficiency. During harvesting, the milky sap produced by the plant, which contains latex and rubber, mixes with the grains, increasing the grain moisture content and contaminating the yield. It also causes significant problems with harvesting machinery [8,9]. *L. serriola* flowers during the grain harvesting season in Australia, and as the flower buds are of the same size as the wheat grain, it is difficult to remove them from the harvested grain, thus heavily reducing the value of the yield due to the presence of these foreign materials [8,10].

*Lactuca serriola* has the ability to withstand environmental stress, especially that of limited water acquisition, due to its root architecture [11]. The ability of *L. serriola* to maintain high water use efficiency under drought conditions makes it an effective coloniser, allowing it to complete its life cycle and to produce a high number of viable seeds even under drought conditions [12]. Its ability to grow through morphological adaptation, as well as physiological and biochemical regulation, even during times of moisture stress, gives this weed species the ability to expand its range under a drying climate. Of interest is that this quality of *L. serriola* makes it a probable source of agriculturally important genes to optimise resource acquisition by cultivated lettuce, which would lead to a reduction in the currently necessary commercial water and fertiliser inputs.

This global literature review focuses on (i) the geographical distribution of *L. serriola*, (ii) the biology of the species (in order to understand its strengths and weaknesses), (iii) its life cycle, as this can identify when the most effective time is to undertake control activities in order to limit its reproduction and further spread, (iv) its seed ecology, this aspect being vital to its management (because the species spreads only via seeds), and (v) current control strategies and their associated problems.

## 2. Distribution

### 2.1. Global Distribution

**Figure 1:**
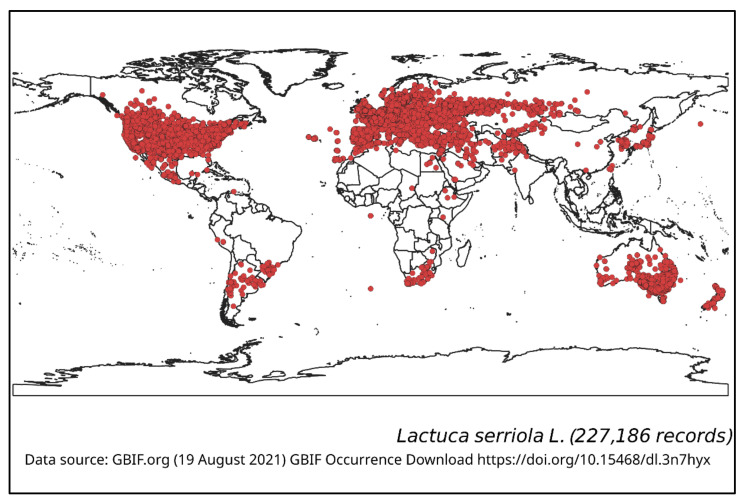
Geographical distribution of *Lactuca serriola*.

*Lactuca serriola* is a Western Eurasian meridional-temperate species, having a synanthropic worldwide distribution whose exact boundary of original distribution area has not been determined [13]. This species has been widely introduced into other regions and it now occurs throughout many continental locations in Europe, Africa, Asia, Australia, a greater part of North America and Central and South America. Though most of the introductions were non-intentional, there are some cases of intentional introduction of this species due to botanical curiosity. The global distribution of *L. serriola* is summarised in Figure 1, which shows both the native range and the introduced area of this species.

From a climatic viewpoint, the northern boundary in the Northern hemisphere of *L. serriola* is limited by cold extreme temperatures. This species is found from lowland to mountain regions, extending up to 1560 m in Switzerland, 1750 m in Turkey, 3100 m in Afghanistan, 3600 m in the Northern Himalayas and 2358 m in the United States of America [1]. Notwithstanding these observed ranges, it is most frequently recorded at elevations of 200–600 m in Europe [5,14], whilst in Great Britain, it is confined to surprisingly low altitudes, only occasionally being seen above 80 m [15,16].

In the Netherlands, *L. serriola* has rapidly become invasive since 1960 in the western parts of the country, and by 2006 was seen to occupy at least 60% of the area [17]. Approximately 16% of *L. serriola* populations were found growing in wet habitats, such as wet ground, ditches, and drains, during a study of the eco geographical distribution in Slovenia [14]. This is in contrast to *L. serriola* populations in Sweden, where they were usually found on gravel or amid stones in dry, sunny areas [14], demonstrating the diverse ecological preferences of *L. serriola*.

*Lactuca serriola* was first brought to Ontario in the late 1890s, and it has since spread over most of Southern Canada via seeds along roadsides, railways, and watercourses. Until the development of reduced tillage practices, which allowed *L. serriola* to thrive in crops, it remained a weed of roadsides and waste ground. Except for Newfoundland, *L. serriola* is now found in most of Canada’s provinces; however, it is rare in Prince Edward Island, Nova Scotia, and New Brunswick [1,18,19]. It is presently most abundant in southern Québec, along the St. Lawrence River in southern Ontario (especially along the Great Lakes), and in the Prairie Provinces and British Columbia’s southern areas [19].

Except for New York, *L. serriola* was found in all of the states visited (16 states) in a research study undertaken in the United States of America [1]. This study observed that *L. serriola* was more commonly found in disturbed habitats, such as the edges of roads and pavements, parking lots and near gas stations, road ditches, and ruderal locations with fertile soil, which is consistent with its position as a weed in North America [20]. This shows that human activities, particularly in transportation corridors, have spread *L. serriola* widely [5,13,21].

### 2.2. Distribution in Australia

*Lactuca serriola* was first recorded in the Upper Hunter region of New South Wales, in 1899 [7], and is now widely naturalised in Australia and New Zealand, being particularly widespread in the eastern and southern parts of Australia (Figure 1) [22]. It is commonly found in south-eastern Queensland, New South Wales, the ACT, Victoria, South Australia and south-western parts of Western Australia, but is less common in Tasmania and the southern parts of the Northern Territory. It is only occasionally recorded in Northern Queensland and other parts of Western Australia (Figure 1).

In Southern New South Wales, this weed has increasingly become a problem in cereal and lucerne pastures, but is also of concern in fallows, gardens, orchards, roadsides and waste lands [7]. While *L. serriola* is commonly a weed in agricultural areas and habitat areas, it is also regarded as an environmental weed in Western Australia, Victoria and the Northern Territory.

*Lactuca serriola* is found growing along with wheat, cereals and pulses in Victoria and South Australia [3]. An increasing incidence of *L. serriola* in dryland crops, such as wheat and chickpeas, was reported in the Wimmera region in Victoria [8]. The emergence pattern of this weed in this region was found to be similar to that in England, with 85% of emergence occurring in autumn and a smaller peak in spring [8]. The weed plant density approached up to 300 m^−2^ shortly after emergence, but then reduced to seven plants m^2^ in fallow and two plants m^2^ in a wheat crop. *L. serriola* plants are more susceptible to dying before stem elongation and had higher mortality when they had competition from a crop compared with no competition. *L. serriola* has spread throughout the Wimmera region, as the production of seeds is mainly after crop harvest, when the only control is by using grazing sheep [8].

### 2.3. Spatial Distribution

Weeds are highly adaptable to new environments due to their variable traits and considerable genetic diversity, which are the key factors in their widespread success and distribution. Invasive species have an advantage over the native flora, as weeds can adapt to a new environment in as little as 20 generations, whilst it may have taken the native flora hundreds or even thousands of years to adapt [23,24]. In this respect, populations that are isolated from each other and exposed to different selective pressures are called ‘spatially varied’ populations, and different biotic and abiotic pressures can cause variations between these spatially varied populations. Such variation in conditions encourages separated populations to select slightly different traits, which over time may result in different phenotypes being expressed [23].

Novotná et al. [25] examined the effect of various soil textures on the morphological features of *L. serriola* achenes from four different countries—the Czech Republic, Germany, the United Kingdom, and the Netherlands. They found that the largest achenes with the longest beaks were found on sandy substrata, whereas the achenes from sandy loam habitats were thinner and had a higher number of ribs. They found that the achene morphology was significantly correlated with the three eco-geographic features of longitude, latitude and the soil texture of the habitats [25]. Significant differences have also been observed in the (i) length of the achene, (ii) width of the achene, (iii) length of pappus bristles, and (iv) pappus area between Slovenian and Swedish populations of *L. serriola* [26]. As *L. serriola* grows in a wide range across several continents, they are expected to have spatial variations due to different types of soil, moisture level and the prevailing weather conditions. However, there is little research to suggest that there are any notable phenotypic variations between overseas populations of *L. serriola*. Furthermore, there has been no genetic analysis to identify the differences in a population’s gene pool, which is unfortunate, because identifying any phenotypic or genotypic differences in regional and international populations could assist with more effective weed management.

The global distribution of *L. serriola*, highlighted in Figure 1, shows how successful this weed is as an invasive species, as it has been dispersed and established across many different environments. However, there is little information regarding any local adaptations of this weed in various countries or regions.

## 3. Biology

### 3.1. Plant Description

**Figure 2:**
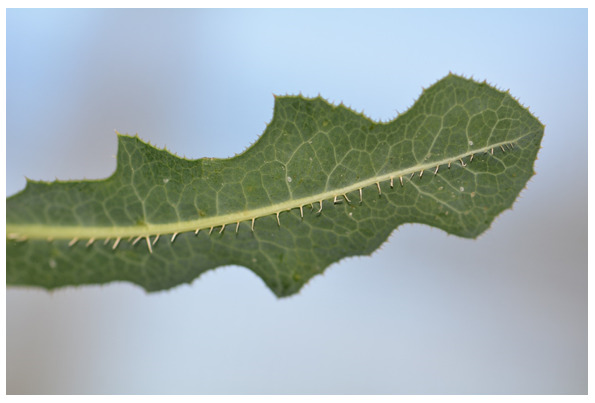
Leaf of *Lactuca serriola*, showing spines on the underside of the midrib and on leaf margins (© Aakansha Chadha).

*Lactuca serriola* is distinguished by its leaves, which have spiny toothed margins and a row of spines on the underside of the leaf and along the midrib, as shown in Figure 2. It is the latter feature that has led to it being alternatively called “prickly lettuce”.

*Lactuca serriola* is an annual plant which reproduces exclusively by seeds and dies after flowering [27]. It is a dicotyledonous plant with rounded cotyledons which are 4–8 mm long with a truncated or indented apex [9,19]. The seedling develops into a basal rosette with a long taproot [6,9]. The rosette leaves are either elongated or oval rounded, widest near the apex, with wavy spiny margins [19,28]. Flowering stems are erect and mostly grow from the centre of the rosette, being spiny near the base. Generally, there is a single stem which elongates from the centre of the rosette, but occasionally, there can be more than one central stem [19,28]. The leaves, stem and the roots exude a milky sap when damaged [10].

Since its stem leaves are held vertically on a north-south plane, *L. serriola* is sometimes known as a “compass plant.” This pattern of non-random leaf orientation may be seen in both the lobed and unlobed varieties of *L. serriola* [29]. Studies on leaf movement in *L. serriola* discovered that fresh leaves twisted at the base due to asymmetric growth and epinastic movement [29]. They proved that leaf twisting is a growth response to direct sunlight, because it was not present in plants cultivated in shaded settings. Because of this leaf orientation, leaves receive maximal radiation in the early morning and late afternoon, while midday radiation loads, leaf temperatures, and vapour pressure are all lowered [30]. Furthermore, the leaf orientation does not change once the leaves are fully expanded, regardless of light exposure [29]. The cauline and the stem leaves alternate and can be 5–25 cm long and are sessile. Some of the leaves, including those that have regrown after mowing or small upper leaves, may lack the spines [10].

Based on the lobes of the leaves, two forms of *L. serriola* occur: forma serriola with deeply lobed cauline leaves and forma integrifolia with unlobed leaves [5,31], and the lobing is genetically controlled by two or three dominant complimentary genes [19]. Some phenotypic variations exist between both the forms, including leaf size, leaf shape, number of spines and wax on the epidermis [29]. Both the leaf forms are mostly found in separate populations; however, they may occur as intermixed [32], and it has been shown that there is no difference in the water use efficiency and leaf surface area between the two leaf forms [29,33].

### 3.2. Life Cycle

The germination of *L. serriola* is largely controlled by temperature and the quality of light [34], and it begins to sprout rapidly under favourable conditions. The main period of germination appears to be in late autumn and early winter with a smaller germination peak in spring [8,27,34]. A few additional seedlings may emerge almost continuously until early summer [27]. As seeds of the same species germinate at different times during the year, they are likely to experience quite different environmental conditions, particularly during the early stages of the life cycle when, in ruderal species, losses are frequently high. Therefore, plants which emerge at different times of the year will have a different life expectancy. This difference in emergence means that plants of *L. serriola* make different contributions to the population as a whole [35]. It is suggested that those which germinate in spring act as an insurance against loss of overwintering seedlings.

Regardless of the germination date, the highest levels of mortality occurred soon after germination, which reduced drastically after the beginning of stem extension [35]. This period is generally considered to be the most hazardous time for most species, since it coincides with the changeover from a dependence on seed reserves to independent assimilation.

*L. serriola* exists over the winter as a seed or rosette and the stem elongation starts in spring, which is the period of the most vegetative growth [19]. The seeds that germinate in spring have a shorter rosette stage, but all the plants flower during spring and early summer, with all the seeds being shed in summer. All plants which emerge between autumn and the following summer die before or during the winter of the second year, irrespective of flowering. This means that the life cycle of the species can vary from winter annual to summer annual [27]. Table 1 summaries the average lifecycle of *L. serriola* in Australia.

The time of seedling emergence had a marked effect on the reproductive output of *L. Serriola* [35]. It is interesting to note that there is a positive correlation between the time a plant spends in the rosette stage and the extent of seeds produced [35]. Seedlings which emerge earliest (in autumn) have been shown to produce approximately 10 times more seeds than those which emerge in spring and summer [34,35].

It was observed that *L. serriola* which had been grown with no competition had 15 lateral branches arising from lower parts of the stem, while most of the plants, when grown in competition with the wheat crop in the Wimmera region of Victoria, Australia, only had a single stem [8]. Branching of the stem frequently occurs after the crop is harvested [8]. It was noted that *L. serriola* reached a height of about 712 mm and had a density of 1.1 plants m^−2^ when growing with wheat crops, whilst the density of *L. serriola* was 7.6 plants m^−2^ on unsown fallow, with the density staying constant throughout the year [8].

Owing to the leaf orientation and root architecture, *L. serriola* can withstand drought [19]. The vertical orientation of the leaf in the north south plane significantly reduces water loss by the plants without affecting the net carbon gain, resulting in high reproductive output [36]. *L. serriola* also produces a deeper root system compared to *Lactuca sativa* (cultivated lettuce) with more laterals near the root tip and 50% of its total root length at the 20 to 80 cm depth. This was confirmed in a greenhouse experiment conducted on *L. serriola* and cultivated lettuce [6,37]. Gallardo et al. [37] also found that drying of the top 20 cm of the soil had no effect on the physiology or growth of *L. serriola*. This was in sharp contrast to the biomass production, leaf water status and photosynthesis of *L. sativa*, all of which were significantly reduced.

### 3.3. Reproduction

*Lactuca serriola* is self-compatible and predominantly self-pollinated [2]. Interspecific hybridization within the genus of *Lactuca* seldom occurs, and the species are highly autogamous [38]. Each flowering stem produces numerous small yellow flower heads or capitula which are 8 to 12 mm in diameter and are borne in panicles at the end of the stem or branches [19]. Each flower head has 10 to 30 pale yellow, ligulate ray flowers or florets which open for only a few hours [19]. Mejías [2] studied four populations from Spain and reported higher fruit set for freely pollinated capitula (94%) compared with bagged flower heads (78%). It was found that plants which emerged in autumn were bigger, flowered earlier and had more seeds compared with plants which emerged in spring [35,39].

Vernalisation of the rosette or the imbibed seeds reduced the time taken from emergence to flowering, and the effect of vernalisation differed with the growth stage [27]. Because the pace of development of plants vernalized as seeds or seedlings was faster than that of plants vernalized as older rosettes, the timing of flowering differed by only a few weeks between plants that emerged in autumn and spring. Once rosettes have been vernalized, they cannot be devernalized. However, the effect of vernalisation can be negated by exposing the seeds to temperatures of 25 to 30 °C in darkness for 1 to 7 days [40]. This cycle of vernalization–devernalization of buried seeds prevents bolting in autumn and allows plants that emerge at different periods of the year to flower at the same time [41].

**Figure 3:**
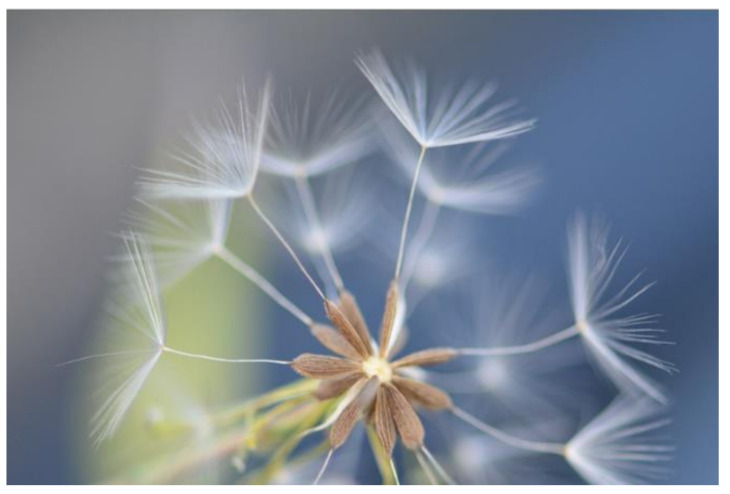
*Lactuca serriola* seed structure showing the brown seed colour, its longitudinal ribs, the bristles near the apex and the pappus (© Aakansha Chadha).

*Lactuca serriola* is spread by achenes (cypselae) and the number of seeds produced is proportional to the plant height [39]. On average, the number of flowers per plant ranged from 250 to 5000, producing between 15 to 22 seeds per capitulum (Figure 3) [8,42]. The growing environment and the competitiveness of the crop can affect the quantity of seeds produced per plant [43]. Over the fruiting period, the number of seeds per capitulum decreased but the weight per individual seed grew in Britain [44] and Israel [45].

In a study carried out by Prince and Carter [44] in five open sites in England which were cultivated and without any crops, it was reported that the number of capitula produced per plant varied from 448 to 6600, which could have more than 100,000 seeds per plant. In Canada, *L. serriola* produced 2200 to 67,000 seeds in a soybean crop, whereas the seed production increased to 87,000 seeds per plant in the non-crop areas adjacent to the fields [39]. *L. serriola* plants without competition in Australia produced an average of 48,000 seeds per plant, whereas the number of seeds decreased as the competition increased [8]. Alcocer-Ruthling et al. [42] reported seed production of 4160 seeds for chlorsulfuron susceptible plants and 4870 seeds for chlorsulfuron-resistant plants which is a negligible difference.

The average seed weight is approximately 0.6 mg, with a range from 0.45 to 0.8 mg [30,42,45]. The colour of the seeds is greyish yellow to brown, with the shape being narrowly oval and flattened and the size being 3 to 4 mm long (Figure 3) [9,19]. Seeds with short fine bristles towards the apex and five to seven longitudinal ribs finish in a beak with 4–5 mm attached pappus (Figure 3) [9,28]. Achenes of both forms of *L. serriola* are distinguished from each other and from achenes of other *Lactuca* spp. by their morphology [16]. In comparison to *L. serriola* f. integrifolia, *L. serriola* f. serriola achenes are shorter, thinner, shorter beaked and have a lower length/width index ratio [25].

*Lactuca serriola* achenes have a pappus (Figure 3) that is invariably monomorphic, consisting of two equal rows of whitish bristles that are longer than the involucral bracts, causing them to be mostly distributed by the wind [28]. There are fine hooks of various sizes and shapes on top of the pappus bristles. Some factors that will affect the dispersal ability of a wind-dispersed (anemochory) seed are seed weight, the size of pappus, and the aerial velocity that the seed can maintain. The seed is light, attached to a pappus that is 4 to 5 mm long, on tall stems, which helps with wind dispersal [18,19,28]. Normally, the seed being released high on the plant helps the seed to gain velocity, allowing the seed to fly in the air for a longer time [46]. Currently, there are limited data on the dispersal ability of *L. serriola*, and it is anticipated that the dispersal ability could vary under different environmental conditions.

## 4. Seed Ecology

### 4.1. Seed Dormancy and Longevity

Seed dormancy is the adaptation of a plant to its habitat to avoid unfavourable environmental conditions and events allowing it to increase its chances for success [47]. Dormancy is a fitness trait related to the establishment, persistence and dispersion of invasive weeds [48]. No primary dormancy has been found in the seeds of *L. serriola*, and they can germinate immediately if the conditions are suitable [34,42]. However, contrasting results were found by Wu et al. [7], wherein up to 75.6% initial dormancy was observed in some of the populations. The viability of seeds is highest during harvest, but it decreases as the seed remains buried for longer [34,42]. According to the study of Alcocer-Ruthling et al. [42], *L. serriola* seeds may remain viable in soil for up to three years, and they also noted that buried seeds lived longer than seeds present on the surface. The viability of surface seed declined to 0% after 12 to 18 months, while that of seed buried at 7.5 to 15 cm was 33 months [42]. It took 80 weeks for buried seeds to reduce viability to 75% [34]. Assessment of field emergence from surface sown seeds indicated that over 90% emergence occurred in the first year, with little emergence in the next two years, after which the soil seed bank was exhausted [7]. The time of year also influenced the viability of *L. serriola* seeds, and the loss of viability was higher in the winter months than during the summer [34]. Additionally, there was no significant difference between the sulfonylurea-susceptible and -resistant biotypes regarding seed longevity [42].

### 4.2. Seed Germination

A temperature range of 10 to 35 °C was found to be conducive to *L. serriola* germination under both constant and alternating temperatures [7,49,50]. However, higher germination rates are obtained at alternating temperatures compared to constant temperatures, as found by Wu et al. [7]. *L. serriola* has not been found to germinate below constant temperatures of 8 °C [34].

*Lactuca serriola* seeds have been found to be non-photoblastic, indicating that when alternate temperature ranges are favourable, light does not have a key role in germination regulation [45,49]. Conversely, Wu et al. [7] found that germination was highly responsive to light treatments. In another study in England, it was found that freshly harvested seeds of *L. serriola* required light when kept at a constant temperature of 15 °C, but not when exposed to alternating temperatures [34]. Contradicting this work is the finding of Jan et al. [51], where their seeds germinated significantly better in the dark than in conditions of 12-hour illumination. These variable responses to light and dark conditions for germination indicate that management should focus on spatial variations among populations.

This weed species can germinate equally well in both acidic and alkaline soils, as experiments with a pH range between 4 and 10 have shown [7,49]. In addition, although *L. serriola* is found in a diverse range of substrata, it was found by Jan et al. [51] that emerging plants had higher germination ability on loess-derived soil than sand-derived podzoic soil, both being measured at the same pH. A significant decline in germination was observed in both spatially varied populations as well as biotypes when conditions of salinity and osmotic stress increased [7,49]. These findings imply that germination of *L. serriola* should be controlled as soon as possible in autumn, spring or early summer when the conditions are favourable for germination. Maximum emergence of seedlings was observed from surface sown seeds and emergence was negatively proportional to burial depth [49]. Emergence declined significantly beyond 4 cm burial depth, and only 0.25% emergence was observed in 10 cm burial depth in one of the biotypes studied [7].

## 5. Management

### 5.1. Chemical Control

Rosettes of *L. serriola* can be managed in autumn or spring by a variety of non-selective herbicides. However, it has been observed that herbicides are not that effective in controlling the weed once the flowering stems start to extend [9,19]. Germinating seedlings can be controlled by pre-emergence application of products containing atrazine, metribuzin, chlorsulfuron, isoxaben, oxyfluorfen, oxadiazon, napropamide or terbacil [10]. Another study by Mikulka and Chodová [50] pointed to the efficacy of herbicides containing amidosulfuron and iodosulfuron as active ingredients, since they provided more than 85% reduction in dry matter content when *L. serriola* plants were treated at the 2–3 leaf stage (Table 2). Once the rosettes are established, they can be controlled in a variety of crops by using 2,4-D, MCPA, metribuzin, dicamba, clopyralid, bromoxylin plus atrazine, linuron, metribuzin, and thifensulfuron-methyl (Table 2) [10]. Seeds that germinate later in the spring generally skip the rosette stage, making them more difficult to control with herbicides, since flowering stems emerge swiftly [9]. Due to the weed’s protracted emergence time, a residual herbicide may be required for season-long control.

### 5.2. Herbicide Resistance

Sulfonylurea-resistant *L. serriola* was first reported in 1987 in a continuous no-till winter wheat crop field in Idaho, USA, and it became one of the dominant weeds in that area in the following five years [53]. Upon discontinuation of the use of sulfonylurea herbicides, the proportion of resistant *L. serriola* decreased by 25–86%, while the area with resistant *L. serriola* increased due to seed movement [54]. As the seeds of *L. serriola* are spread by the wind, it is possible that resistance to herbicides could spread rapidly from one site to another. The mechanism of ALS-inhibiting herbicide resistance in *L. serriola* was determined to be the result of a resistant form of ALS [55]. In the population collected from Idaho, the mutation that contributes to resistance is in Domain A of ALS, where proline residue was modified to histidine [56]. Moreover, in the year 2007, resistance to 2,4-D, dicamba, and MCPA was reported in Washington, USA in cereal crops [57,58].

In South Australia, resistance to sulfonylurea was first reported in 1994 in *L. serriola* where it has evolved resistance to the ALS inhibiting herbicides chlorsulfuron, flumetsulam, metosulam, metsulfuron-methyl and triasulfuron [58]. The resistant populations have cross resistance to other ALS-inhibiting herbicides and have been identified to be moderately resistant to triazolopyrimidine and imidazolinone herbicides [4]. The resistance in the South Australian populations was owing to a modification of ALS, wherein proline residue was modified to threonine [4]. The rapid selection of sulfonylurea resistant *L. serriola* in South Australia is probably the result of (i) a relatively high frequency of initial resistance in untreated populations, (ii) the effective control of *L. serriola* at low rates of sulfonylurea herbicides, and (iii) the persistence of sulfonylureas in the soil due to the alkaline soils in the area [4]. In Victoria, a state of Australia, resistance to chlorsulfuron and metsulfuron-methyl has been recorded in spring barley and wheat, and also to glyphosate in fallow [58].

### 5.3. Alternate Methods of Control

As *L. serriola* has a long tap root system [6], it is easy to manually remove the plant. Grubbing will kill the plant, and if implemented before the seed set, it can greatly reduce the seedbank. The downside of this technique is that it can only be used for small areas and not where the weed is widespread and dense, as it is a time-consuming process. Tillage can easily control seedlings and rosettes of *L. serriola* during non-crop periods [9]. Because this species possesses a taproot, superficial tillage may be just as beneficial as deep tillage in this situation. Tillage works best when the soil is dry, the air is warm, and the relative humidity is low, allowing the plants to wilt quickly. Tillage should be practiced in the autumn and early spring when *L. serriola* rosettes are easier to control [9]. Grazing of rosettes is another option for the control of *L. serriola* for large infestations, since sheep and goats can effectively reduce its population. However, young plants appear to be toxic and can cause pulmonary emphysema in cattle feeding on them [10]. Rosettes are not effectively controlled by mowing, because their leaves are too close to the soil surface to be cut. Plants that have been mowed after stem elongation generate additional stems or lateral branches which bear flowers [10], and therefore mowing of *L. serriola* is not an option for long-term control. Given the mobility of *L. serriola* seeds, it has been suggested that controlling plants near the boundary of a seeded crop field could help limit the generation and spread of a significant number of seeds into the region [9,39].

Members of the Brassicaceae family contain glucosinolates, a class of secondary plant metabolites which have been tested as a bioherbicide for *L. serriola* and have been found to have potential [59]. This suggests that bioherbicides could be explored further, to be used in aspects of vegetable farming or as a part of an integrated weed management strategy.

Seedlings and rosettes of *L. serriola* are easily managed by competition. In a study conducted by Weaver et al. [39] in a winter wheat crop, it was found that the winter wheat harvest significantly interrupted the flowering of *L. serriola,* with only 25% to 30% of the plants surviving the harvest, and later when flowering, producing less than 4000 seeds in untreated stubble. The administration of non-selective herbicides is possible when winter wheat is rotated with spring crops or summer fallow. Control during planting resulted in the lowest in-crop populations of *L. serriola* and the lowest crop yield losses [39].

### 5.4. Integrated Weed Management

It has been widely suggested that to reduce the dependence on the use of herbicides, integrated weed management, which is a holistic weed control approach developed by integrating different weed control methods (chemical, physical and cultural), should be introduced worldwide for the effective control of *L. serriola.* According to the literature, the suggested method of management and control is to firstly manually remove small and/or isolated infestations, ensuring the entire plant is removed, especially the taproot. Next, a combination of herbicides should be used during the seedling or rosette stage of *L. serriola*, as it is a most effective practice. However, research has shown that *L. serriola* can increase in abundance after effective control the previous year, indicating that a one-off management strategy is insufficient in controlling this weed [60]. Attention should be given to reducing the input of seeds into the soil seed bank, especially as this species produces a large number of highly mobile and fertile seeds. In this respect, more research is required to identify how competition can be best utilised to control the emergence and growth of *L. serriola.*

## 6. Conclusions

The ability of *L. serriola* to establish itself in a wide range of environmental conditions in numerous countries is key to its effective establishment and propagation [61,62]. *L. serriola* is capable of expanding rapidly into new locations and becoming dense in the existing locations due to its invasive and aggressive traits. This species has the capacity to invade many areas in Australia, as it can germinate in a range of temperatures when sufficient moisture is available. As *L. serriola* seeds are wind-borne, herbicide resistance can spread rapidly to become more pronounced in the near future, especially as the area of herbicide resistance has increased in South Australia. It is agreed that future research is required to use integrated weed management to control *L. serriola* and not rely solely on alternative herbicides to control the spread of current herbicide resistance.

Many examples of spread have apparently been assisted by *L. serriola’s* staggered germination which enables some plants to avoid the effects of herbicides commonly applied to cereal and grain legume crops [52]. Future studies should consist of seedling ecology experiments, since mortality of this species is highest soon after germination. Additionally, strategies which minimise weed survival while maximizing irrigation proficiency for the crop needs to be implemented to reduce the impact of *L. serriola* on crops.

Finally, the short seed bank persistence of *L. serriola* recommends that it is highly possible to control this species with a dedicated management program for 3–4 years where no new seed should be added into the soil seedbank. Since more than 90% of surface-placed seeds emerge in the first year after burial, early management is especially important in the first year of infestation. However, weeds along fence lines and along roadsides should be managed carefully, as the light-weight seeds generated by *L. serriola* plants in nearby non-cropping areas can quickly reinfest clean paddocks.

## Figures and Tables

**Table 1:** Average lifecycle of *Lactuca serriola* in Australia.

	Autumn			Winter			Spring			Summer		
Life Cycle	Mar	Apr	May	Jun	Jul	Aug	Sep	Oct	Nov	Dec	Jan	Feb
Germination		O	Y	Y	Y	Y	Y	O	O			
Rosette stage			Y	Y	Y	Y	Y					
Active growth			O	O	O	O	Y	Y	Y	Y	Y	Y
Flowering	O							Y	Y	Y	Y	Y
Seeding	O									Y	Y	Y

Note: “Y” indicates regular growth patterns and “O” indicates occasional growth.

**Table 2:** Herbicides tested for the control of *Lactuca serriola*.

Herbicide Used	Crop	Rate	Time of Application	*Lactuca serriola* Control
^1^ Pyrasulfotole + bromoxynil (Huskie)	Wheat	13.5 oz/acre	NA	95% [9]
^1^ Florasulam + MCPA (Orion)	Wheat	17 oz/acre	NA	93% [9]
^1^ Clopyralid + fluroxypyr (WideMatch)	Wheat	16 oz/acre	NA	92% [9]
^1^ Metribuzin	Wheat	12 oz/acre	NA	81% [9]
^1^ Fluroxypyr (Starane Ultra)	Wheat	6.4 oz/acre	NA	80% [9]
^1^ Bromoxylin (Buctril)	Wheat	24 oz/acre	NA	76% [9]
^1^ Diuron (Karmex DF)	Wheat	16 oz/acre	NA	73% [9]
^1^ Dicamba	Wheat	4 oz/acre	NA	71% [9]
^1^ MCPA amine 4	Wheat	16 oz/acre	NA	71% [9]
^1^ Prosulfuron (Peak)	Wheat	0.38 oz/acre	NA	66% [9]
^1^ Carfentrazone-ethyl (Aim)	Wheat	1.0 oz/acre	NA	63% [9]
^2^ Chlorsulfuron	Wheat	0.02 kg a.i/ha	0 WAS	7.3 plants/m^2^ [52]
^2^ Chlorsulfuron	Wheat	0.02 kg a.i/ha	6 WAS	0.9 plants/m^2^ [52]
^3^ Chlorsulfuron	Wheat	0.01 kg a.i/ha	8 WAS	1.3 plants/m^2^ [52]
^3^ Chlorsulfuron	Wheat	0.02 kg a.i/ha	8 WAS	0.7 plants/m^2^ [52]
^3^ Chlorsulfuron	Wheat	0.04 kg a.i/ha	8 WAS	0 plants/m^2^ [52]
^4^ Metribuzin + methabenzthiazuron	Wheat	0.10 + 0.42 kg a.i/ha	4 WAS	3.3 plants/m^2^ [52]
^2^ Metribuzin + methabenzthiazuron	Wheat	1.10 + 0.42 kg a.i/ha	6 WAS	5.4 plants/m^2^ [52]
^3^ Metribuzin + methabenzthiazuron	Wheat	0.05 + 0.21 kg a.i/ha	8 WAS	0 plants/m^2^ [52]
^3^ Metribuzin + methabenzthiazuron	Wheat	1.10 + 0.42 kg a.i/ha	8 WAS	0 plants/m^2^ [52]
^3^ Metribuzin + methabenzthiazuron	Wheat	0.21 + 0.84 kg a.i/ha	8 WAS	0 plants/m^2^ [52]
^4^ Ametridione	Wheat	1.0 kg a.i/ha	4 WAS	1.0 plants/m^2^ [52]
^4^ Methabenzthiazuron	Wheat	0.6 kg a.i/ha	4 WAS	31.2 plants/m^2^ [52]
^2^ Methabenzthiazuron	Wheat	0.6 kg a.i/ha	6 WAS	7.4 plants/m^2^ [52]
^2^ MCPA (amine form)	Wheat	0.42 kg a.i/ha	8 WAS	18.8 plants/m^2^ [52]
^2^ MCPA (amine form)	Wheat	0.42 kg a.i/ha	10 WAS	1.2 plants/m^2^ [52]
^4^ MCPA (amine form)	Wheat	0.42 kg a.i/ha	14 WAS	0.5 plants/m^2^ [52]
^2^ Dicamba	Wheat	0.14 kg a.i/ha	10 WAS	0.3 plants/m^2^ [52]
^4^ Dicamba	Wheat	0.14 kg a.i/ha	14 WAS	0.6 plants/m^2^ [52]
^2^ 2,4-D amine	Wheat	0.35 kg a.i/ha	10 WAS	0.3 plants/m^2^ [52]
^4^ 2,4-D amine	Wheat	0.35 kg a.i/ha	14 WAS	0.3 plants/m^2^ [52]
^5^ Oxyfluorfen	Chickpeas	0.12 kg a.i/ha	Pre	6.27 plants/m^2^ [52]
^5^ Oxyfluorfen	Chickpeas	0.24 kg a.i/ha	Pre	10 plants/m^2^ [52]
^5^ Oxyfluorfen	Chickpeas	0.36 kg a.i/ha	Pre	1.74 plants/m^2^ [52]
^6^ Terbutryne	Chickpeas	0.28 kg a.i/ha	Post-pre	16.01 plants/m^2^ [52]
^5^ Terbutryne	Chickpeas	1.0 kg a.i/ha	Pre	11.35 plants/m^2^ [52]
^5^ Cyanazine	Chickpeas	2.0 kg a.i/ha	Pre	9.22 plants/m^2^ [52]
^6^ Methabenzthiazuron	Chickpeas	0.38 kg a.i/ha	Post	4.98 plants/m^2^ [52]
^7^ Methabenzthiazuron	Chickpeas	1.0 kg a.i/ha	Post-pre	22.66 plants/m^2^ [52]
^5^ Methabenzthiazuron	Chickpeas	1.75 kg a.i/ha	Pre	9.33 plants/m^2^ [52]
^5^ Ametridione	Chickpeas	1.0 kg a.i/ha	Post-pre	0.19 plants/m^2^ [52]
^6^ Metribuzin	Chickpeas	0.4 kg a.i/ha	Post-pre	3.0 plants/m^2^ [52]
^6^ Metribuzin + methabenzthiazuron	Chickpeas	0.10 + 0.42 kg a.i/ha	Post-pre	5.34 plants/m^2^ [52]
^6^ Prometryne	Chickpeas	0.5 kg a.i/ha	Post-pre	21.52 plants/m^2^ [52]
^7^ Prometryne	Chickpeas	0.55 kg a.i/ha	Post-pre	13.64 plants/m^2^ [52]
^6^ MCPA (Sodium salt)	Chickpeas	0.12 kg a.i/ha	Post	3.78 plants/m^2^ [52]
^6^ MCPA (Sodium salt)	Chickpeas	0.22 kg a.i/ha	Post	1.91 plants/m^2^ [52]
^6^ MCPA (Sodium salt)	Chickpeas	0.34 kg a.i/ha	Post	1.52 plants/m^2^ [52]
^8^ Tribenuron (Granstar 75 WG)	NA	10.85 g a.i/ha	2–3 leaves stage	32.9% [50]
^8^ Florasulam + 2,4-D (Mustang)	NA	3.12 + 150 g a.i/ha	2–3 leaves stage	25.4% [50]
^8^ Picloram + clopyralid (Galera)	NA	16.75 + 66.75 g a.i/ha	2–3 leaves stage	29.8% [50]
^8^ Picloram + clopyralid (Galera)	NA	26.8 + 106.8 g a.i/ha	2–3 leaves stage	28.2% [50]
^8^ 2,4-D (Esteron 60)	NA	676.8 g a.i/ha	2–3 leaves stage	19.7% [50]
^8^ Clopyralid (Lontrel 300)	NA	90 g a.i/ha	2–3 leaves stage	31.0% [50]
^8^ Amidosulfuron (Grodyl 75 WG)	NA	22.5 g a.i/ha	2–3 leaves stage	9.1% [50]
^8^ Fluroxypyr (Starane 250 EC)	NA	200 g a.i/ha	2–3 leaves stage	27.5% [50]
^8^ Iodosulfuron-methyl + mefenpyr-diethyl (Husar)	NA	7.5 + 18 g a.i/ha	2–3 leaves stage	15.3% [50]
^8^ Picolinafen + cyanazine (Outlook WG)	NA	120 + 480 g a.i/ha	2–3 leaves stage	33.2% [50]
^8^ Amidosulfuron + Iodosulfuron-methyl + mefenpyr-diethyl (Sekator)	NA	10 + 2.5 + 25 g a.i/ha	2–3 leaves stage	12.8% [50]

Note: WAS = weeks after sowing; Pre = pre-sowing; Post-pre = post-sowing, pre-emergence; Post = post emergence, 6 weeks after sowing; NA = not available. ^1^ Rate is in oz/acre as this information has been obtained from an American source. Visual control scale is from 0 to 100% with 0% being no visible damage and 100% being plant death. ^2^ Control for these treatments had 28.7 plants/m^2^. ^3^ Control for these treatments had 4.2 plants/m^2^. ^4^ Control for these treatments had 139.1 plants/m^2^. ^5^ Control for these treatments had 18.43 plants/m^2^. ^6^ Control for these treatments had 25.41 plants/m^2^. ^7^ Control for these treatments had 18.31 plants/m^2^. ^8^ Control is represented as % of untreated control in shoots of *Lactuca serriola*.

## Data Availability

Not applicable.

## References

[1] Lebeda A., Doležalová I., Novotná A. (2012). Wild and Weedy *Lactuca* Species, Their Distribution, Ecogeography and Ecobiology in USA and Canada. Genet. Resour. Crop. Evol..

[2] Mejías J.A. (1994). Self-Fertility and Associated Flower Head Traits in the Iberian Taxa of *Lactuca* and Related Genera (*Asteraceae*: *Lactuceae*). Plant Syst. Evol..

[3] Llewellyn R., Ronning D., Clarke M., Walker S., Ouzman J. (2016). Impact of Weeds on Australian Grain Production: The Cost of Weeds to Australian Grain Growers and the Adoption of Weed Management and Tillage Practices.

[4] Preston C., Stone L.M., Rieger M.A., Baker J. (2006). Multiple Effects of a Naturally Occurring Proline to Threonine Substitution within Acetolactate Synthase in Two Herbicide-Resistant Populations of *Lactuca serriola*. Pestic. Biochem. Physiol..

[5] Lebeda A., Doležalová I., Křístková E., Mieslerová B. (2001). Biodiversity and Ecogeography of Wild *Lactuca* spp. in Some European Countries. Genet. Resour. Crop. Evol..

[6] Jackson L. (1995). Root Architecture in Cultivated and Wild Lettuce (*Lactuca* spp.). Plant Cell Environ..

[7] Wu H., Asaduzzaman M., Shephard A., Hopwood M., Ma X. (2020). Germination and Emergence Characteristics of Prickly Lettuce (*Lactuca serriola* L.). Crop. Prot..

[8] Amor R. (1986). Incidence and Growth of Prickly Lettuce (*Lactuca serriola*) in Dryland Crops in the Victorian Wimmera. Plant Prot. Q..

[9] Burke I.C., Lyon D.J. Integrated Management of Prickly Lettuce in Wheat Production Systems.

[10] Weaver S., Downs M. Prickly Lettuce. http://www.omafra.gov.on.ca/english/crops/facts/03-041.htm.

[11] Johnson W., Jackson L., Ochoa O., Van Wijk R., Peleman J., Clair D.S., Michelmore R.W. (2000). Lettuce, a Shallow-Rooted Crop, and *Lactuca serriola*, Its Wild Progenitor, Differ at QTL Determining Root Architecture and Deep Soil Water Exploitation. Theor. Appl. Genet..

[12] Chadha A., Florentine S.K., Chauhan B.S., Long B., Jayasundera M. (2019). Influence of Soil Moisture Regimes on Growth, Photosynthetic Capacity, Leaf Biochemistry and Reproductive Capabilities of the Invasive Agronomic Weed; *Lactuca serriola*. PLoS ONE.

[13] Lebeda A., Dolezalová I., Feráková V., Astley D. (2004). Geographical Distribution of Wild *Lactuca* Species (*Asteraceae*, *Lactuceae*). Bot. Rev..

[14] Doležalová I., Lebeda A., Křístková E. (2001). Prickly Lettuce (*L. serriola* L.) Germplasm Collecting and Distribution Study in Slovenia and Sweden. Plant Genet. Resour. Newsl..

[15] Prince S.D., Carter R.N., Dancy K.J. (1985). The Geographical Distribution of Prickly Lettuce (*Lactuca Serriola*): II. Characteristics of Populations Near its Distribution Limit in Britain. J. Ecol..

[16] Carter R.N., Prince S.D. (1985). The Geographical Distribution of Prickly Lettuce (*Lactuca serriola*). I. A General Survey of Its Habitats and Performance in Britain. J. Ecol..

[17] Hooftman D.A.P., Oostermeijer J.G.B., Den Nijs J.C.M. (2006). Invasive Behaviour of *Lactuca serriola* (*Asteraceae*) in The Netherlands: Spatial Distribution and Ecological Amplitude. Basic Appl. Ecol..

[18] Frankton C., Mulligan G. (1987). Weeds of Canada (Revised). Publication 948.

[19] Weaver S., Downs M. (2003). The Biology of Canadian Weeds. 122. *Lactuca serriola* L. Can. J. Plant Sci..

[20] United States Department of Agriculture (USDA) *Lactuca serriola* L. Prickly Lettuce. https://plants.usda.gov/home/plantProfile?symbol=LASE.

[21] Lebeda A., Doležalová I., Křístková E., Dehmer K., Astley D., Van de Wiel C., Van Treuren R. (2007). Acquisition and Ecological Characterization of *Lactuca serriola* L. Germplasm Collected in the Czech Republic, Germany, the Netherlands and United Kingdom. Genet. Resour. Crop. Evol..

[22] Agriculture Victoria Prickly Lettuce. http://vro.agriculture.vic.gov.au/dpi/vro/vrosite.nsf/pages/sip_salt_prickly_lettuce.

[23] Oduor A.M., Leimu R., Kleunen M. (2016). Invasive Plant Species Are Locally Adapted Just as Frequently and At Least as Strongly as Native Plant Species. J. Ecol..

[24] Prentis P., Wilson J., Dormontt E., Richardson D., Lowe A. (2008). Adaptive Evolution in Invasive Species. Trends Plant Sci..

[25] Novotná A., Doležalová I., Lebeda A., Kršková M., Berka T. (2011). Morphological Variability of Achenes of Some European Populations of *Lactuca serriola* L. Flora.

[26] Eva K., Lebeda A., Novotná A., Doležalová I., Berka T. (2014). Morphological Variation of *Lactuca serriola* L. Achenes as a Function of Their Geographic Origin. Acta Bot. Croat..

[27] Prince S.D., Marks M.K., Carter R.N. (1978). Induction of Flowering in Wild Lettuce (*Lactuca serriola* L.). New Phytol..

[28] Uva R.H., Neal J.C., DiTomaso J.M. (1997). Weeds of the Northeast.

[29] Werk K., Ehleringer J. (1984). Non-Random Leaf Orientation in *Lactuca serriola* L. Plant Cell Environ..

[30] Werk K., Ehleringer J. (1986). Effect of Nonrandom Leaf Orientation on Reproduction in *Lactuca serriola* L. Evolution.

[31] Prince S.D., Carter R.N. (1977). Prickly Lettuce (*Lactuca serriola* L.) in Britain. Watsonia.

[32] Oswald P. (2000). Historical Records of *Lactuca serriola* L. and *L. virosa* L. in Britain, with Special Reference to Cambridge Shire (vc 29). Watsonia.

[33] Werk K., Ehleringer J. (1985). Photosynthetic Characteristics of *Lactuca serriola* L. Plant Cell Environ..

[34] Marks M.K., Prince S.D. (1982). Seed Physiology and Seasonal Emergence of Wild Lettuce *Lactuca Serriola*. Oikos.

[35] Marks M., Prince S. (1981). Influence of Germination Date on Survival and Fecundity in Wild Lettuce *Lactuca serriola*. Oikos.

[36] Werk K., Ehleringer J. (1986). Field Water Relations of a Compass Plant, *Lactuca serriola* L. Plant Cell Environ..

[37] Gallardo M., Jackson L., Thompson R. (1996). Shoot and Root Physiological Responses to Localized Zones of Soil Moisture in cultivated and Wild Lettuce (*Lactuca* spp.). Plant Cell Environ..

[38] Feráková V. (1977). Genus Lactuca L. in Europe.

[39] Weaver S., Cluney K., Downs M., Page E. (2006). Prickly Lettuce (*Lactuca serriola*) Interference and Seed Production in Soybeans and Winter Wheat. Weed Sci..

[40] Marks M.K., Prince S.D. (1979). Induction of Flowering in Wild Lettuce (*Lactuca serriola* L.). II. Devernalization. New Phytol..

[41] Prince S.D., Marks M.K. (1982). Induction of Flowering in Wild Lettuce (*Lactuca serriola* L.); III, Vernalization-Devernalization Cycles in Buried Seeds. New Phytol..

[42] Alcocer-Ruthling M., Thill D.C., Shafii B. (1992). Seed Biology of Sulfonylurea-Resistant and-Susceptible Biotypes of Prickly Lettuce (*Lactuca serriola*). Weed Technol..

[43] Lu Y.Q. (2006). The Spread of Herbicide Resistance in Lactuca serriola at a Landscape Scale.

[44] Prince S.D., Carter R.N. (1985). The Geographical Distribution of Prickly Lettuce (*Lactuca Serriola*): III. Its Performance in Transplant Sites Beyond Its Distribution Limit in Britain. J. Ecol..

[45] Gutterman Y. (1992). Maturation Dates Affecting the Germinability of *Lactuca serriola* L. Achenes Collected from a Natural Population in the Negev Desert Highlands. Germination under Constant Temperatures. J. Arid. Environ..

[46] Burrows F., Murray D.R. (1986). The Aerial Motion of Seeds, Fruits, Spores and Pollen. Seed Dispersal.

[47] Baskin C., Baskin J. (2014). Seeds: Ecology, Biogeography, and Evolution of Dormancy and Germination.

[48] Presotto A., Poverene M., Cantamutto M. (2014). Seed Dormancy and Hybridization Effect of the Invasive Species, *Helianthus annuus*. Ann. Appl. Biol..

[49] Chadha A., Florentine S., Chauhan B.S., Long B., Jayasundera M., Javaid M.M., Turville C. (2019). Environmental Factors Affecting the Germination and Seedling Emergence of Two Populations of an Emerging Agricultural Weed: Wild Lettuce (*Lactuca serriola*). Crop. Pasture Sci..

[50] Mikulka J., Chodová D. (2003). Germination and Emergence of Prickly Lettuce (*Lactuca serriola* L.) and Its Susceptibility to Selected Herbicides. Plant Soil Environ..

[51] Jan K., Małgorzata H., Paweł H. (2012). The Effect of Soil Environment on Germination and Emergence of Prickly Lettuce (*Lactuca serriola* L.). Acta Agrobot..

[52] Amor R. (1986). Chemical Control of Prickly Lettuce (*Lactuca serriola*) in Wheat and Chick-Peas in the Victorian Wimmera. Plant Prot. Q..

[53] Mallory-Smith C.A., Thill D.C., Dial M.J. (1990). Identifilcation of Sulphonylurea Herbicide-Resistant Prickly Lettuce (*Lactuca serriola*). Weed Technol..

[54] Alcocer-Ruthling M., Thill D.C., Mallory-Smith C. (1992). Monitoring the Occurrence of Sulfonylurea-Resistant Prickly Lettuce (*Lactuca serriola*). Weed Technol..

[55] Eberlein C.V., Guttieri M.J., Mallory-Smith C.A., Thill D.C., Baerg R.J. (1997). Altered Acetolactate Synthase Activity in ALS-Inhibitor Resistant Prickly Lettuce (*Lactuca serriola*). Weed Sci..

[56] Guttieri M.J., Eberlein C.V., Mallory-Smith C.A., Thill D.C., Hoffman D.L. (1992). DNA Sequence Variation in Domain A of the Acetolactate Synthase Genes of Herbicide-Resistant and-Susceptible Weed Biotypes. Weed Sci..

[57] Burke I.C., Yenish J.P., Pittmann D., Gallagher R.S. (2009). Resistance of a Prickly Lettuce (*Lactuca serriola*) Biotype to 2, 4-D. Weed Technol..

[58] Heap I. The International Survey of Herbicide Resistant Weeds. http://www.weedscience.org/Pages/Species.aspx.

[59] Handiseni M., Brown J., Zemetra R., Mazzola M. (2011). Herbicidal Activity of Brassicaceae Seed Meal on Wild Oat (*Avena fatua*), Italian Ryegrass (*Lolium multiflorum*), Redroot Pigweed (*Amaranthus retroflexus*), and Prickly Lettuce (*Lactuca serriola*). Weed Technol..

[60] Merriam A.B., Malone J., Gill G., Preston C. (2021). Can Rotations Improve Management of Herbicide-Resistant Annual Sowthistle (*Sonchus oleraceus*) and Prickly Lettuce (*Lactuca serriola*) in Lentil Production Systems of Southern Australia?. Weed Technol..

[61] D’Andrea L., Broennimann O., Kozlowski G., Guisan A., Morin X., Keller-Senften J., Felber F. (2009). Climate Change, Anthropogenic Disturbance and the Northward Range Expansion of *Lactuca serriola* (*Asteraceae*). J. Biogeogr..

[62] D’Andrea L., Meirmans P., van de Wiel C., Guadagnuolo R., van Treuren R., Kozlowski G., den Nijs H., Felber F. (2017). Molecular Biogeography of Prickly Lettuce (*Lactuca serriola* L.) Shows Traces of Recent Range Expansion. J. Hered..

